# Post-Polio Syndrome in a Primary Care Setting: A Case Report

**DOI:** 10.7759/cureus.29361

**Published:** 2022-09-20

**Authors:** Ammar Khan, Anna Virani

**Affiliations:** 1 Physical Medicine and Rehabilitation, Herbert Wertheim College of Medicine, Florida International University, Miami, USA; 2 Family Medicine, Herbert Wertheim College of Medicine, Florida International University, Miami, USA

**Keywords:** poliomyelitis, poliovirus, salk vaccine, motor neuron disease, post-polio syndrome

## Abstract

Post-Polio Syndrome (PPS) is a sequela of poliovirus infection that causes weakness in previously infected polio patients years after the initial infection. The diagnosis is one of exclusion and entails the following: 1) a prior episode of poliomyelitis with residual motor neuron function loss, 2) a period of at least 15 years or more after the acute onset of polio with neurologic and functional stability, and 3) a gradual onset of new weakness and abnormal muscle fatigability that has persisted for at least one year. While the exact etiology is unknown, the prevalence of PPS has increased as patients who have previously survived polio are getting older. In this report, we discuss a patient presenting to his primary care provider for evaluation of worsening lower extremity weakness over the course of the past three years. In addition to general characteristics of PPS, we will review the use of electromyography (EMG)/nerve conduction studies and imaging for evaluation. This report will also review prevention methods with vaccinations and identify potential treatment regimens including aerobic exercise and medications ranging from tricyclic antidepressants (TCAs) to dopamine agonists. The goal of this paper is to not only shine a light on PPS in general, but to show how social determinants i.e., economic stability, healthcare access and quality of health may affect the diagnosis of uncommon conditions.

## Introduction

Poliomyelitis is an infection of the motor neurons in the anterior horn of the spinal cord and brain stem caused by the poliovirus. Poliovirus is a neurotropic enterovirus that, while eradicated in most parts of the world, is still endemic throughout South Asia, mainly found in Pakistan and Afghanistan [1]. The aim of this report is to bring awareness to Post-Polio Syndrome (PPS), a potential sequela of the poliovirus that is becoming more prevalent given that previously infected individuals are surviving to older ages [2]. PPS diagnosis requires the following: 1) a prior episode of poliomyelitis with residual motor neuron function loss, 2) a period of at least 15 years or more after the acute onset of polio with neurologic and functional stability, and 3) a gradual onset of new weakness and abnormal muscle fatigability that has persisted for at least one year. The diagnosis of PPS is also one of exclusion, meaning that this diagnosis may only be considered after having ruled out more common causes of muscle weakness [3]. The main cause of PPS is still widely unknown. Multiple studies have looked at the potential causes including reactivation of a latent form of the virus, progression of motor neuron degeneration, and induction of autoimmunity causing inflammation [4,5]. However, these investigations have all been found to be inconclusive to this point. With all of this in mind, it is easy to see why this would become a challenge to diagnose in underserved areas. Their social determinants of health may be a barrier to proper workup for this diagnosis. Specifically, economic status and healthcare access need to be considered [6].

## Case presentation

The patient is a 61-year-old male, with a past medical history of poliovirus infection, benign prostate hypertrophy, and hyperlipidemia, seen in a primary care setting. He presented with worsening weakness in his lower extremities for the past three months. At the age of two years while growing up in Pakistan, the patient was diagnosed with polio. His family recounts that he had flu-like symptoms at the onset of his illness (fever and fatigue) along with a significant lower extremity muscle weakness. He states that this weakness was very noticeable in his legs when he was younger but improved when he was approximately five years old. The patient noticed his lower extremity weakness returning when he was approximately 58-years-old. He felt that the weakness has been slow in onset and increased in intensity over time. He denies any inciting events. He has experienced several falls over the past three years which prompted his use of a cane. He has been ambulating with a cane since then. He denies incurring any specific trauma to his lower back or having any pain in that area. The patient reports difficulty getting up from a chair and sitting back down. He also noticed that he is fatigued after standing for long periods of time, despite using his cane. He denies any pain, fever, chills, nausea, vomiting, diarrhea, constipation, loss of sensation, bowel or bladder problems, paresthesia, chest pain, and shortness of breath. He denies recent infections or illnesses. He has never been hospitalized and the only procedure he underwent was a colonoscopy at age 50, which was reportedly normal. He does not take any prescribed, over-the-counter, or herbal medications and has no known allergies. He denies the use of tobacco products, alcohol, and illicit drugs. He is sexually active with his wife and has never had a previous diagnosis of any sexually transmitted infections. His family history is significant for type II diabetes in his mother and unspecified heart disease in his father. On the physical exam, inspection revealed thin extremities without any obvious deformities. He did not feel any pain on palpation of any of his muscles or joints. However, palpation shows decreased tone in his lower extremities. There is significant atrophy of the gastrocnemius and quadriceps bilaterally. The tone is normal in the upper extremities. His sensation is intact throughout his upper and lower extremities. The patient did not reveal any abnormalities in the range of motion (ROM) for his upper extremities. He is unable to actively range his lower extremities in any effective manner. He has 1/5 strength in bilateral hip flexion, knee flexion, and knee extension. His reflexes are intact in his upper extremities and absent in his lower extremities (Achilles and patellar). Cranial nerves are grossly intact. The remainder of the physical exam is normal. The patient’s lab work from a prior visit had demonstrated a normal complete blood count (CBC), comprehensive metabolic panel (CMP), thyroid stimulating hormone (TSH), hemoglobin (Hb)A1c, and slightly elevated low-density lipoprotein (LDL) cholesterol. The patient’s lumbar X-ray from a prior visit for the same concern showed prominent levoscoliosis, mild degenerative disc disease, and reverse spondylolisthesis of the L3-L4 region.

The patient was given a referral to neurology and an interim T2 weighted MRI to evaluate other potential causes of his weakness. A referral to PM&R (Physical Medicine and Rehabilitation) was also ordered for proper evaluation through an EMG/nerve conduction study. He has been doing home exercises to help strengthen his lower extremities and working to obtain local health insurance through financial assistance programs. Unfortunately, at this time, the patient is having trouble completing these referrals and evaluations due to a number of social determinants. For this patient, his financial insecurity, lack of access to healthcare, and lack of transport are his biggest obstacles at the moment. The patient declined to provide pictures of his legs and only brought the report of his lumbar X-ray so no images could be provided for the case at this time.

## Discussion

Based on a review of current literature, PPS occurs in approximately 20-30% of polio survivors [7]. It appears to be secondary to an absence of reinnervation of the anterior horn cells caused by the initial polio infection. There are multiple theories investigating the causes of PPS.

One of the theories resembles the same pathophysiology as shingles. It is believed to be a reactivation of the dormant virus causing further degeneration of motor neurons and preventing reinnervation. The virus does not reactivate in everyone diagnosed with polio. Patients receiving the inactivated form of the vaccine through intramuscular injections have no reported cases of polio either, eliminating the concern for PPS. However, patients vaccinated using the attenuated live oral polio vaccine (OPV) have recorded active cases of polio due to the vaccination. Providers should keep PPS in their differential in these patients if they begin to have symptom recurrence after a diagnosis of polio secondary to OPV. This is because an infection of polio after the vaccine means an active form of the virus has infected the anterior horn cells. Given that OPV is a weakened form of the virus, it is a reasonable assumption that the risk of polio disease is low. However, that does not mean that there is zero risk of polio after receiving the vaccine. That is why those with polio symptoms after OPV administration should be treated as polio patients who became infected through the natural, non-iatrogenic route. For this same reason, we must monitor the patients experiencing polio, from any etiology, for PPS [7]. Other theories include an autoimmune condition affecting the motor neurons triggered by previous poliomyelitis. The evidence supporting this includes the presence of elevated protein and oligoclonal bands seen in CSF in some of the PPS patients [1]. The last major theory surrounding PPS is a progression of motor neuron degeneration from the original infection. All three of these theories of mechanism lack sufficient evidence for the medical community to confidently attribute them to the causes of PPS [8]. 

Our patient has not undergone specific testing to rule out more common diagnoses such as amyotrophic lateral sclerosis (ALS), polyneuropathies, etc. [9]. Therefore, he could not be formally diagnosed with PPS at this time. In addition, the lab work ordered in the clinic was normal aside from elevated LDL cholesterol. When taking into consideration the patient’s history, physical exam findings, and progression of symptoms, it is not unreasonable to move PPS higher up on the differential. The recommendation for the patient was for further evaluation at another institution with the necessary equipment to complete nerve conduction studies, EMG, and a T2 weighted MRI [10,11]. The MRI is used to rule out compression of the spinal cord. The lack of evidence of sensory neuropathy allowed us to rule down the likelihood that his weakness is a result of his spondylolisthesis. In addition, the spondylosis noted in the X-ray would most likely present with some form of back pain with potential radiation of the pain down the legs. However, the patient is strictly complaining of weakness.

A wide differential should be utilized when examining a patient with suspected PPS. The first diagnosis to consider is amyotrophic lateral sclerosis (ALS). The patient’s symptoms do not appear to line up with the classic presentation of ALS given that he does not have a constant linear progression of symptoms and the weakness has not progressed past his legs in the past three years. Further investigation with nerve conduction studies to evaluate for nerve potentials is required to truly rule out ALS [1]. There is also a lack of upper motor neuron signs in this patient which decreases the likelihood of ALS as the diagnosis. The other condition high on the differential is lumbar spondylolisthesis causing compression of the motor nerve roots. Patients can experience weakness in their legs, but they also typically have sensory symptoms. They also experience relief with lumbar flexion and worsening their symptoms with lumbar extension [12]. However, this patient did not experience any alteration in his symptoms with any positional changes. The last diagnosis to consider on the differential is another form of demyelinating polyneuropathy. As mentioned in the evidence above, this differential was ruled down due to the presentation of the patient’s case. However, like ALS, a nerve conduction study is needed to properly evaluate nerve function. The patient’s lab values and clinical presentation make myositis or hypothyroidism less likely than PPS. Timely diagnosis is critical for patients with suspected PPS, as the therapy listed below prevents further atrophy and weakness. Without the proper intervention, patients could continue to deteriorate while providers search for an answer within the negative imaging and lab work.

The management of PPS includes aerobic intensity exercise along with work-stop physical exercise programs. The evidence of these programs is supported by several small uncontrolled studies due to the small patient population and lack of funding and research in this field [9]. There is no evidence that pyridostigmine and steroids, such as prednisone, have any efficacy in the muscle weakness seen in PPS [1]. In addition, intravenous immunoglobulin (IVIG) has been shown to have no improvement in the management of the strength or fatigue of PPS patients [1]. Similar studies have looked at a number of medications to address different symptoms of PPS. For instance, some patients with PPS have been seen to have Restless Leg Syndrome. These patients improved with dopamine agonists for symptom management. Other symptoms that may arise in PPS include but are not limited to respiratory dysfunction, bulbar symptoms, and joint instability.

**Figure 1:**
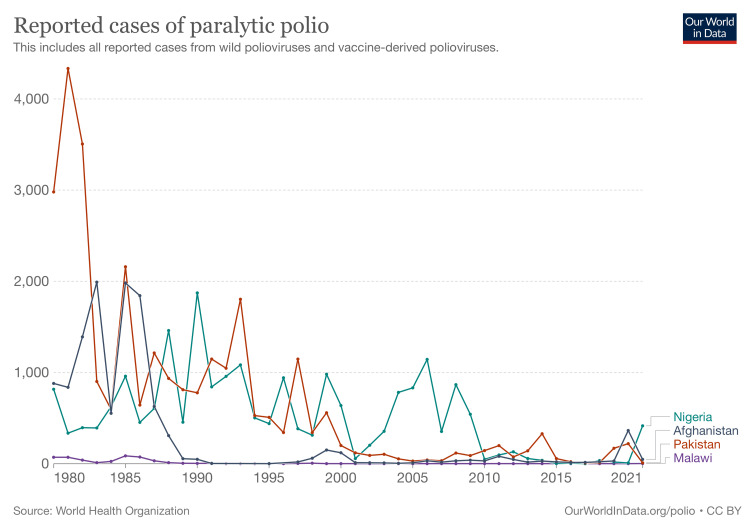
Reported cases of paralytic polio Reproduced with permission from Our World in Data under the Creative Commons BY license, 2022

It is important to globally promote and secure access to inactivated polio vaccines for all conditions, including the Salk vaccine (the inactivated version of the polio vaccine) [10,11]. That being said, Figure 1 shows us that the number of cases in the past can also represent a growing population of patients that may experience lasting consequences of the disease such as PPS [13]. The effectiveness of three doses of the polio vaccine is 99-100% according to the latest studies conducted by the CDC [7]. Therefore, providers should promote all of their patients to receive the inactivated polio vaccine as long as the patients do not have any contraindications. Prevention of polio subsequently leads to the prevention of PPS. The public should not only focus on the acute condition aspects of any viral illness but on the risks of suffering from the long-term effects of these same viruses. This case should not only serve as a catalyst for more research to help a growing PPS patient population, maintaining the differential for at-risk patients, but also for the necessity for vaccines as means of protection against the disease in the short and long term.

## Conclusions

This case brings to light a population that remains vulnerable to complications from polio despite the virtual eradication of the parent condition in the United States. PPS causes muscle weakness and atrophy in individuals which greatly impacts their quality of life particularly when they remain without timely and proper diagnosis and treatment. If providers are unaware of the prevalence of PPS, they may inadvertently neglect a growing population of patients. While a wide differential is still necessary and the diagnosis is one of exclusion, it still remains crucial to keep this diagnosis in the back of providers' minds so providers can properly care for their patients.

Not only is it important to identify PPS to accurately care for these patients, but it is equally important to promote preventive medicine through early diagnosis, isolation, and vaccines. The vaccination debate has peaked with the COVID-19 pandemic. People’s views are more polarized today than ever have been primarily due to the dissemination of a significant amount of misinformation. As mentioned earlier, vaccination with the inactivated form is a strong method of preventing polio infection and its sequelae. The management of PPS is strongly centered around physical and occupational therapy. While there do not appear to be any medications on the market to target the weakness and fatigue seen in PPS, there are medications for the associated symptoms. 
